# TWEAK increases angiogenesis to promote diabetic skin wound healing by regulating Fn14/EGFR signaling

**DOI:** 10.1111/jocd.16486

**Published:** 2024-08-21

**Authors:** Ying‐jie Zhu, Hu‐lin Chen, Jing‐kai Huang, Xin‐jie Cai, Bang‐le Zhan

**Affiliations:** ^1^ Department of Dermatology Southern University of Science and Technology Hospital Shenzhen Guangdong China; ^2^ Department of Dermatology Guangdong Women and Children Hospital Guangzhou Guangdong China

**Keywords:** angiogenesis, diabetes mellitus, fibroblast growth factor‐inducible 14/epidermal growth factor receptor signaling, tumor necrosis factor‐like weak inducer of apoptosis, wound healing

## Abstract

**Objective:**

Tumor necrosis factor‐like weak inducer of apoptosis (TWEAK), a member of tumor necrosis factor superfamily, can bind to fibroblast growth factor‐inducible 14 (Fn14) receptor and stimulate angiogenesis. The interaction between epidermal growth factor receptor (EGFR) and endothelial growth factor (EGF) leads to EGFR signal transduction and promotes angiogenesis. The objective of this study was to explore whether TWEAK participated in the diabetic skin wound healing by regulating Fn14/EGFR signaling.

**Methods:**

Human umbilical vein endothelial cells (HUVECs) were treated with 35 mmol/L d‐glucose and classified into the Control Group, High Glucose (HG) Group and HG + TWEAK Group. Then, the TWEAK expression and the proliferation, migration and tubule formation of HUVECs were detected, respectively. In vivo experiment, the diabetic model was established by injecting streptozotocin (STZ, 50 mg/kg) into male BALB/c mice. On the back of successfully modeled diabetic mice, a full‐thickness skin wound of 6 mm diameter was formed. Then, the mice were randomly assigned into three groups: Blank Group, Phosphate Buffer Saline (PBS) Group, and TWEAK Group. Subsequently, expression levels of TWEAK, Fn14, EGFR and vascular endothelial growth factor (VEGF)‐A were measured, and the CD31 expression in the wounded skin tissue of mice was checked by immunohistochemistry staining.

**Results:**

The expression level of TWEAK in HUVECs of HG Group decreased significantly, as well as the viability, migration, and tubule formation of cells. After over‐expression of TWEAK, the cell viability, migration, and tubule formation abilities of HUVECs recovered remarkably. In vivo, the wound healing rate of diabetic mice was raised, the neovascularization was increased, and the CD31 expression in the wounded tissue was obviously upregulated after injection with recombinant TWEAK antibody.

**Conclusion:**

TWEAK stimulates angiogenesis and accelerates the wound healing of diabetic skin by regulating Fn14/EGFR signaling.

## 1 INTRODUCTION

Diabetes mellitus (DM), a severe chronic metabolic condition, has a major global impact on public and clinical health. Currently, there are approximately 463 million people diagnosed as DM worldwide.^1^ DM may lead to multiple complications, including diabetic wounds, diabetic retinopathy, and diabetic nephropathy.^2^ Chronic non‐healing diabetic wound is one of the critical complications of DM, causing serious clinical and financial burdens.^2^ The diabetic wounds are characterized by fibroblast dysfunction, abnormal inflammatory response, epithelialization, and a lack of chemokine production and angiogenesis.^2^ Impaired angiogenesis is a major reason for delayed wound healing in diabetic patients.^3^ Therefore, promoting angiogenesis is essential to promote the recovery of diabetic vascular complications.^4^

Tumor necrosis factor‐like weak inducer of apoptosis (TWEAK) and its homologous receptor fibroblast growth factor‐inducible 14 (Fn14) are members of the tumor necrosis factor superfamily.^5^ TWEAK is widely expressed in monocytes, dendritic cells, and natural killer (NK) cells, with macrophages/monocytes being the main source of soluble TWEAK (sTWEAK) in inflammatory tissues.^6^ It is reported that Fn14 is expressed in skin resident cells such as dermal fibroblasts, dermal microvascular endothelial cells, and keratinocytes.^7^ It is closely associated with cell growth and adhesion migration.^8^ TWEAK stimulates angiogenesis and promotes cell proliferation and migration. Besides, Fn14 possibly participates in TWEAK‐induced endothelial cell proliferation and angiogenesis.^9^

Research indicates that appropriate TWEAK/Fn14 signaling is required for the healing of damaged tissues.^7^ Skin tissue remodeling and collagen synthesis are influenced by TWEAK/Fn14 signaling‐regulated downstream cytokines or receptors, including epidermal growth factor receptor (EGFR). EFGR is primarily expressed in the epithelial cells, promoting cell proliferation, migration, and survival.^10^ In addition, the interaction between EGFR and its major ligand endothelial growth factor (EGF) leads to EGFR signal transduction and promotes angiogenesis.^11^ Other studies have pointed out that TWEAK/Fn14 signals can significantly regulate vascular endothelial growth factor (VEGF), with TWEAK able to induce tumor angiogenesis by stimulating Fn14 and inducing VEGFA directly and indirectly.^12^

Collectively, TWEAK and Fn14 interact with each other, thereby participating in angiogenesis, fibrosis, and tissue remodeling. Such interaction is vital in the wound healing process. We speculate that TWEAK/Fn14 signaling may play a role in the skin healing of DM. Therefore, this study was designed to explore the therapeutic effect of TWEAK on skin healing in diabetic mice and its underlying mechanisms.

## 2 MATERIALS AND METHODS

### 2.1 Cell grouping and drug intervention

Human umbilical vein endothelial cells (HUVECs) were cultured in a Dulbecco's Modified Eagle Medium (DMEM, Gibco, USA) containing 10% fetal bovine serum (FBS, Gibco, USA), 100 μg/mL streptomycin, and 100 U/mL penicillin. The cell culture was performed in a cell incubator at 37°C with 5% CO_2_. The cultured HUVECs^13^, ^14^ were separated into the following three groups: Control Group: HUVECs cultured under normal conditions; High Glucose (HG) Group: HUVECs treated with 35 mmol/L d‐glucose for 24 h. HG + TWEAK Group: HUVECs treated with 200 ng/mL recombinant TWEAK for 24 h after intervention with 35 mmol/L d‐glucose.

### 2.2 Diabetic mice modeling

BALB/c male mice aged 8 weeks were housed under specific pathogen‐free (SPF) conditions with a controlled diet to ensure consistent nutritional status. The mice were given streptozotocin (STZ, 50 mg/kg) through intraperitoneal injection for 5 days to induce diabetes. Two weeks later, the blood glucose levels of the mice was measured. If the blood glucose was higher than 16.7 mM, the diabetic mice modeling was considered successfully established. Subsequently, a full‐thickness skin wound of 6 mm diameter was created on the back of the successfully modeled diabetic mice by excision. The health and infection status of the mice were monitored regularly throughout the experiment to ensure that no infections or health complications influenced the results.

### 2.3 Animal grouping and drug intervention

Successfully modeled mice were randomly divided into three groups (15 mice per group)^15^, ^16^, ^17^: Blank Group: mice did not receive further treatment; Phosphate Buffer Saline (PBS) Group: 100 μL PBS was injected at four sites around the wound every 3 days (25 μL PBS per site); and TWEAK Group: 100 μL recombinant TWEAK (20 μg/mL, prepared in PBS) was injected at 4 sites around the wound every 3 days.

### 2.4 Real‐time fluorescence quantitative PCR (qRT‐PCR) analysis

Total RNA from cells was extracted according to mRNA extraction kit (K157002, Thermo Fisher Scientific, USA). The concentration and purity of the extracted RNA were determined based on the A260/A280 ratio. Reverse transcription was performed for the synthesis of cDNA single strand using the reverse transcription kit (R312‐01, Nanjing Vazyme Biotech Co., Ltd.). Appropriate amounts of cDNA were used as templates for PCR, with GAPDH as an internal reference. Next, the TWEAK mRNA gene expression in each cell group was examined by fluorescence quantitative PCR amplification using (qRT‐PCR) analyzer (R312‐01, Vazyme Biotech Co., Ltd) with the following parameters: pre‐denaturation for 3 min at 95°C, denaturation for 10 s at 95°C, denaturation for 30 s at 58°C; for a total of 39 cycles; extension for 5 s at 65°C. After completion of the reaction, the amplification curve and dissolution curve of samples were determined. The method of 2^−△△CT^ was adopted for analysis and calculation of the results.

### 2.5 Western blot analysis

The protocol of the protein extraction kit (024C1007, Shanghai Epizyme Biomedical Technology Co., Ltd) was followed to extract the total protein from cells and tissues. Then, the protein was subject to 12% polyacrylamide gel electrophoresis. Subsequently, the protein was placed onto a polyvinylidene fluoride (PVDF) membrane. The membrane was then sealed using 5% skimmed milk. Next, an incubation was performed with primary antibodies overnight at 4°C. The primary antibodies used included TWEAK (ab37170, Abcam), Fn14 (ab109365, Abcam), EGFR (ab52894, Abcam), and VEGFA (ab46154, Abcam). On the next day, the primary antibodies were washed away, and the second antibody (abs20002ss, Abcam) was added for another 2 h of incubation with the membrane on the shaker. Then, the second antibody was rinsed, and enhanced chemiluminescence (ECL) solution (sb‐wb012, Shanghai ShareBio Technology Co., Ltd.) was evenly added to the membrane for a few minutes of reaction. After that, multifunctional imaging system (Vilber, Fusion FX5 Spectra, France) was employed to detect the strips. Finally, Image‐J software (National Institutes of Health, USA) was adopted to measure the gray value of the strip and analyze the results.^18^

### 2.6 Determination of cell viability

The HUVEC viability was analyzed according to the operational steps of MTT assay kit (M1020, Beijing Solarbio Technology Co., Ltd.). Briefly, HUVECs were introduced into 96‐well plates at a density of 2 × 10^3^ cells per well (200 μL/well). Upon treatment with HG or HG + TWEAK, the cells were incubated for 0, 24, 48, and 72 h. Next, 20 μL MTT assay reagent was added, and the cells were incubated under the condition of 37°C, 5% CO_2_. Four hours later, the culture was discontinued, followed by a careful aspiration of the culture supernatant in the well. Subsequently, cells in each well were mixed with 150 μL dimethyl sulfoxide (DMSO), and the mixtures were shaken for 10 min to completely melt the crystals. Finally, the absorbance value at 490 nm was read using a microplate reader (Tecan, Switzerland), and the results were recorded and analyzed.

### 2.7 Scratch test

HUVECs at logarithmic phase were collected, digested, and prepared into cell suspension. Upon counting, the cell suspension at a concentration of 1 × 10^6^ cells/mL was seeded into a six‐well plate with a volume of 2 mL per well. For every group, three duplicate wells were established. The cell culture was performed in an incubator under the condition of 37°C, 5% CO_2_. After formation of monolayer cells, a vertical scratch was created by a 10‐μL pipet tip, and the floated cells were fully rinsed away using PBS. Subsequently, the treated cells were split into three groups (HG + TWEAK Group, HG Group, and Control Group), and were treated as indicated. Next, an inverted fluorescent microscope was employed to observe and photograph each group of cells from the upper, middle, and lower visions randomly selected. Lastly, the scratch areas were evaluated using the Image‐J software (National Institutes of Health, USA).

### 2.8 Tubule formation test

Matrigel was added into a 24‐well plate at a volume of 200 μL/well. Then, HUVECs at the logarithmic phase were collected and seeded into this 24‐well culture plate with a density of 1 × 10^5^ cell/well. The cells were grouped into the HG + TWEAK Group, HG Group, and Control Group, and were treated as indicated. Each group had three duplicate wells. Next, the cell tubule formation was examined under a 100 × inverted fluorescent microscope. Five areas were randomly selected for each group to image the tubule formation. The experiment was repeated for 3 times.

### 2.9 Wound area calculation

Photos of the mice wound in the Blank, PBS, and TWEAK Groups were shot at the 0th, 4th, 6th, 8th and 12th day, respectively. The wound healing rate was calculated after recording the wound areas. Wound healing rate (%) = (*W*_0_−*W*_n_)/*W*_0_ × 100. *W*_0_ represented the wound area at the 0 day, Wn indicated the wound area of the 4th, 6th, 8th, and 12th day.

### 2.10 Hematoxylin–eosin staining (H&E)

According to H&E kit (DH0006, LEAGENE) steps, the tissue was sectioned, baked, dewaxed, hydrated, stained by hematoxylin–eosin, mounted by neutral gum, and dried. Then, histomorphology changes of mice in three groups at the 12th day were observed using the microscope (Leica DM2500, Germany) and photographed.

### 2.11 Immunohistochemistry (IHC) staining

IHC staining was carried out following the steps of the corresponding kit (SAP‐9100, Beijing ZSGB Biotechnology Co., Ltd.). Briefly, the tissue sections were subject to dewaxing, hydrating, antigen repairing and endogenous peroxisome blocking, followed by incubation with the primary antibody CD31 (ab28364, Abcam) overnight at 4°C. On the next day, HRP‐conjugated goat anti‐rabbit IgG (D110058, Shanghai Sangon Biotechnology Co., Ltd.) was supplemented for another hour of incubation at ambient temperature. Then, the sections were stained with hematoxylin, developed using DAB reagent (ZLI‐9017, Beijing ZSGB Biotechnology Co., Ltd.), dehydrated, transparent and sealed. The IHC staining results were inspected by two senior histopathologists. Positive cells were present with light yellow or brown cell membrane/cytoplasm. The results were analyzed in Image‐Pro Plus v6.0 software (Media Cybemetic, USA).

### 2.12 Statistical method

A power analysis was performed to support the justification of the sample size. We anticipated a medium effect size (Cohen's *d* = 0.5), a significance level (α) of 0.05, and a desired power (1−*β*) of 0.95. Using G*Power software, the power analysis indicated that a minimum of 14 mice per group is required. Therefore, 15 mice per group were used in this study.

Experimental data of each group were analyzed using SPSS23.0 statistics software. The data were presented as mean ± standard deviation (SD). One‐way ANOVA followed by Tukey's test was performed for multiple comparisons. To control for type I error inflation due to multiple comparisons, we employed the Bonferroni correction method. Two‐way ANOVA was employed to analyze the interaction between two independent variables. Independent samples *t*‐tests were used to compare the data between two groups. There were three iterations for each experiment at least. *p* < 0.05 indicated a statistical significance.

## 3 RESULTS

### 3.1 Downregulation of TWEAK in high glucose‐induced HUVECs

**FIGURE 1:**
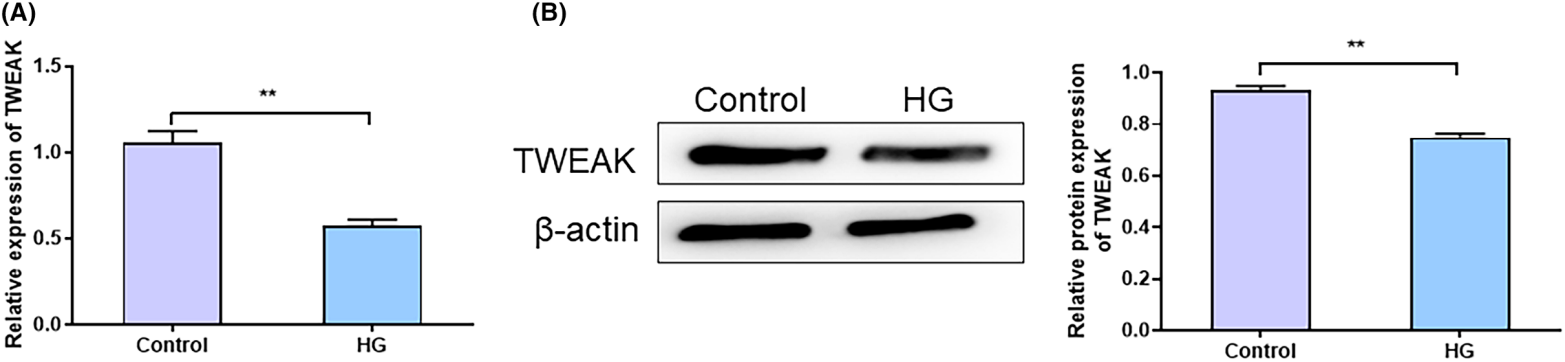
Downregulation of TWEAK in high glucose‐induced HUVECs. (A) qRT‐PCR to detect the expression level of TWEAK mRNA in the Control Group and HG Group. (B) Western blot to detect the protein expression level of TWEAK in the Control and HG Groups. **p < 0.01 versus Control Group. qRT‐PCR, real‐time fluorescence quantitative PCR; TWEAK, tumor necrosis factor‐like weak inducer of apoptosis; HG, high glucose.

The TWEAK expression in HG‐induced HUVECs was detected. qRT‐PCR results demonstrated a significant reduction in TWEAK expression in the HG Group compared to the Control Group (*t* = 10.85, *p* = 0.0004) (Figure 1A). Similarly, Western blot analysis showed a notable decrease in TWEAK protein expression in the HG Group compared to the Control Group (*t* = 14.24, *p* = 0.0001) (Figure 1B).

### 3.2 TWEAK enhances the proliferation, migration, and tubule formation of HG‐induced HUVECs

**FIGURE 2:**
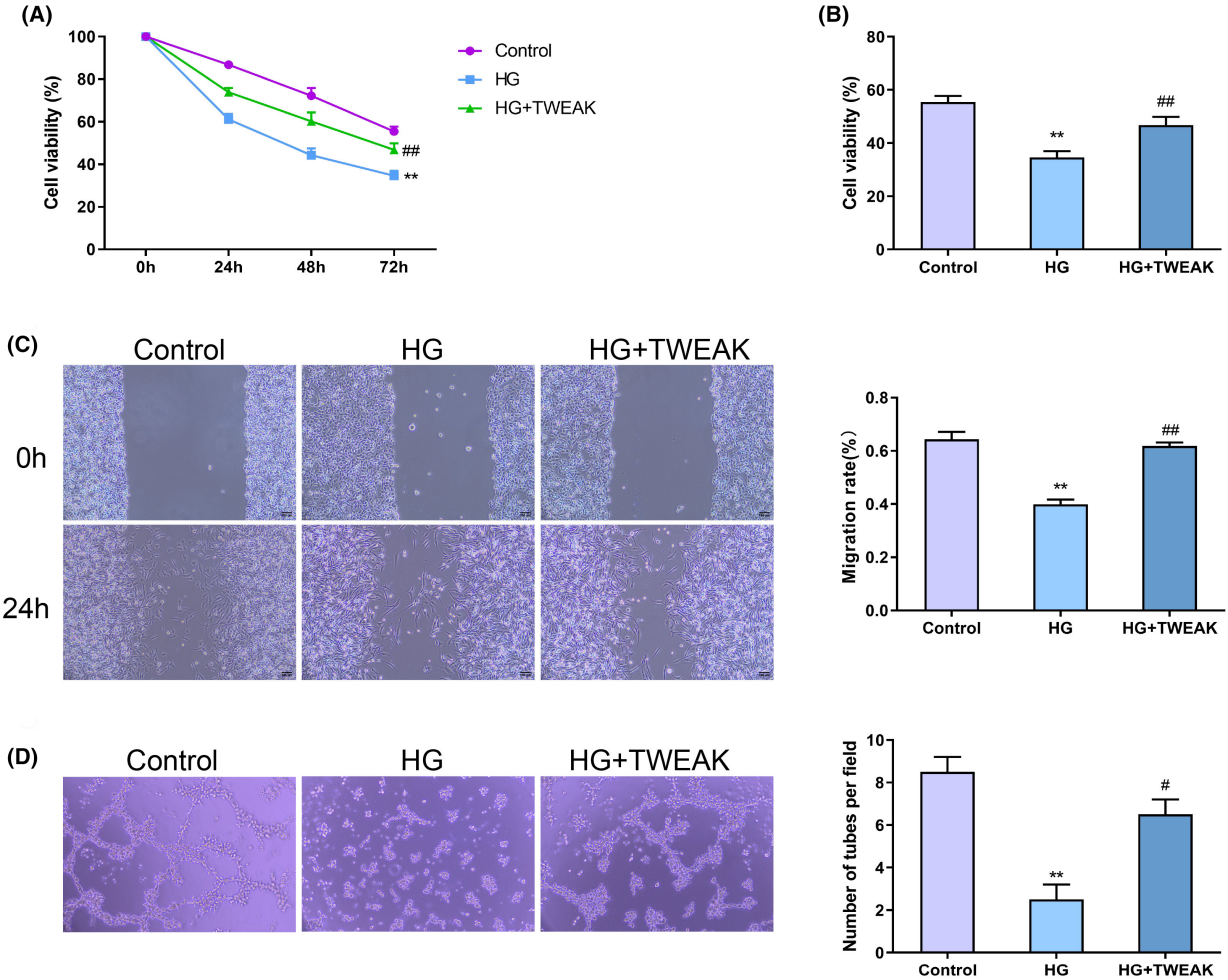
TWEAK enhances the proliferation, migration and tubule formation ability of HG‐induced HUVECs. (A) MTT assay was used to detect the viability of HUVECs at 0, 24, 48, and 72 h. (B) Cell viability of HUVECs in the Control Group, HG Group, and HG + TWEAK Group. (C) Scratch experiment was conducted to check the migration ability of HUVECs in the Control Group, HG Group, and HG + TWEAK Group. (D) Tubule formation experiment was employed to determine the tubule formation ability of HUVECs in the Control Group, HG Group, and HG + TWEAK Group. **p < 0.01 versus Control Group; ^#^ *p* < 0.05, ^##^ *p* < 0.01 versus HG Group. HUVECs, human umbilical vein endothelial cells; HG, high glucose; TWEAK, tumor necrosis factor‐like weak inducer of apoptosis.

MTT assay showed that the cell viability of each group progressively decreased over time, according to a two‐way ANOVA (F (6, 24) = 20.17, *p* < 0.00001) (Figure 2A). Further analysis revealed that, relative to the Control Group, the cell viability of HUVECs in the HG Group notably decreased compared to the Control Group (*p* = 0.0002, Cohen's *d* = 9.57), whereas the HG + TWEAK Group showed a significant increase in viability in contrast to the HG Group (*p* = 0.0147, Cohen's *d* = 3.18) (Figure 2B). In addition, there was a significant difference in migration ability between the TWEAK‐treated group and the control groups, according to a one‐way ANOVA (*F* (2,3) = 87.09, *p* = 0.0022). Tukey's post hoc tests indicated that the migration ability of HUVECs in the HG Group was much higher than that in the Control Group (*p* = 0.0026, Cohen's *d* = 10.49). However, the migration ability of HUVECs was obviously higher in the HG + TWEAK Group compared with the HG Group (*p* = 0.0035, Cohen's *d* = −14.20) (Figure 2C). As illustrated in Figure 2D, there was a significant difference in tubule formation between the TWEAK‐treated group and the control groups, according to a one‐way ANOVA (*F* (2,3) = 37.33, *p* = 0.0076). Tukey's post hoc tests indicated that the HG Group had much lower tubule formation ability than the Control Group (*p* = 0.0070, Cohen's *d* = 8.49), while the HG + TWEAK Group exhibited significantly increased tubule formation relative to the HG Group (*p* = 0.022, Cohen's *d* = −5.66). The above results indicated that TWEAK could improve the ability of cell proliferation, migration, and tubule formation in HG‐induced HUVECs.

### 3.3 TWEAK promotes the wound healing of diabetic mice

**FIGURE 3:**
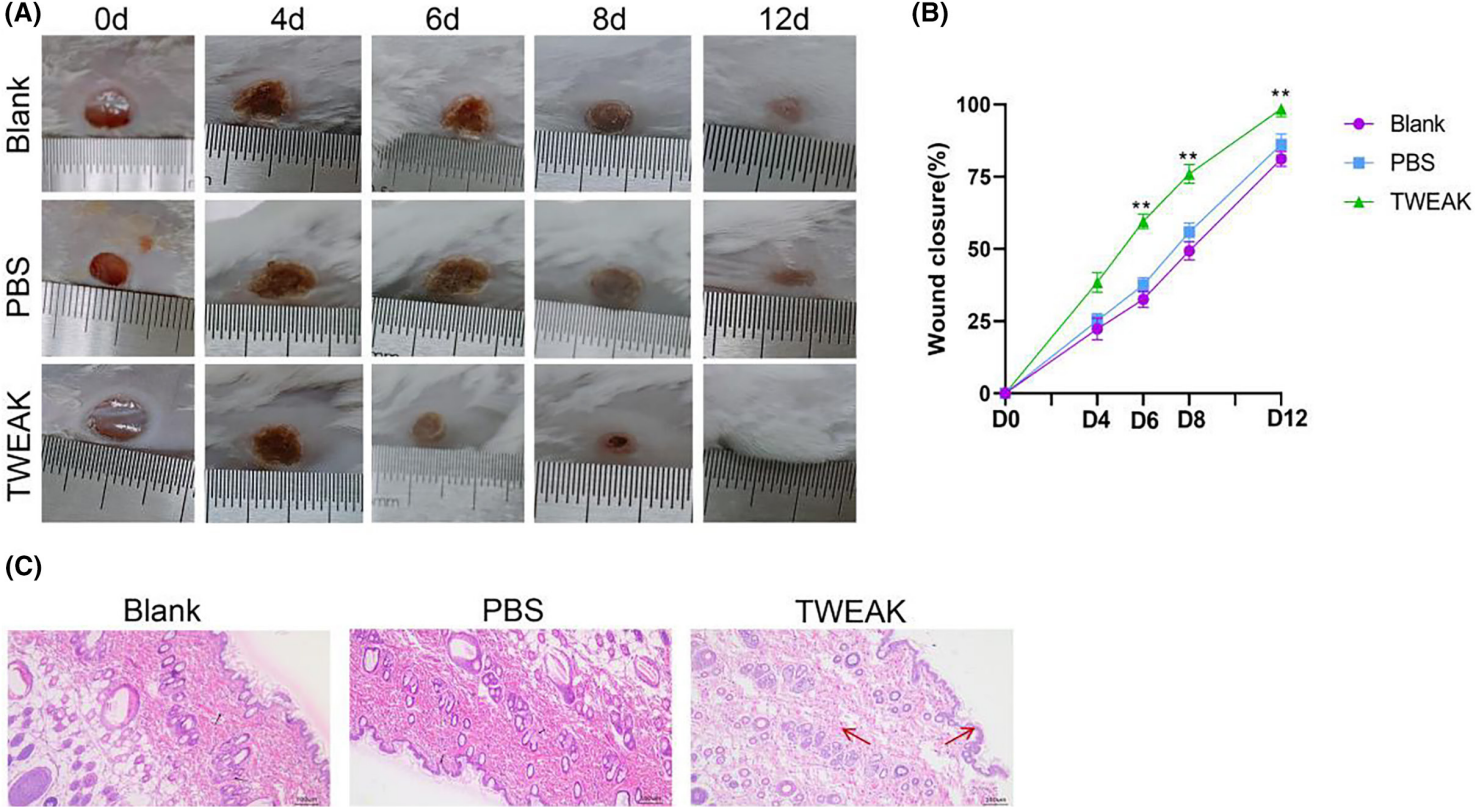
TWEAK promotes the wound healing of diabetic mice. (A) The mice wound photos of the Blank, PBS, and TWEAK Groups were taken on the 0th, 4th, 6th, 8th, and 12th day. (B) The wound closure rate of mice in the Blank, PBS, and TWEAK Groups was shown. (C) H&E staining showed the histomorphology changes of mice in the Blank Group, PBS Group, and TWEAK Group on day 12. **p < 0.01 versus PBS Group. PBS, phosphate buffer saline; TWEAK, tumor necrosis factor‐like weak inducer of apoptosis; H&E, hematoxylin–eosin.

Diabetic mice model was further employed for in vivo experimental verification. The result revealed that the progressive wound healing improved over time in all groups (Figure 3A). On the 0th day, no significant difference in the wound healing rate was observed among the three groups (*p* > 0.05). On Day 4, the wound healing rate of the TWEAK Group was slightly higher compared to the PBS Group and Blank Group without significant difference (*p* > 0.05). On Days 6, 8, and 12, there was a significant difference in wound healing rate between the TWEAK group and PBS group, according to a two‐way ANOVA (*F*(8,30) = 13.65, *p* < 0.0001) (Figure 3B). H&E staining revealed, a higher degree of re‐epithelialization and dermal regeneration in the TWEAK Group compared to the PBS and Blank Groups, accompanied with the thin keratinized and spinous layers in the new epidermis as well as increased neovascularization (Figure 3C). The data suggested that TWEAK could promote the wound healing of diabetic mice.

### 3.4 Local TWEAK promotes angiogenesis at the wound of diabetic mice

**FIGURE 4:**
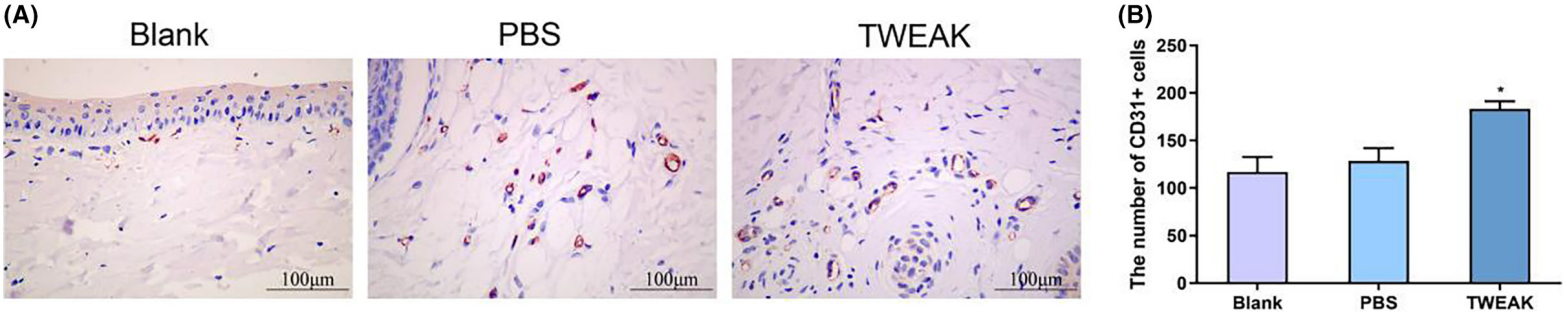
Local TWEAK promotes angiogenesis at the wound of diabetic mice. (A) Immunohistochemistry (IHC) staining assay was used to detect CD31 level in the wound skin tissue of mice in the Blank Group, PBS Group, and TWEAK Group on day 12. (B) The quantity of CD31 positive cells was calculated. *p < 0.05 versus PBS Group. PBS, phosphate buffer saline; TWEAK, tumor necrosis factor‐like weak inducer of apoptosis.

To determine whether TWEAK promoted angiogenesis of the wounded site, IHC staining for endothelial marker CD31 was performed. The results showed significantly increased CD31 expression in the TWEAK Group as opposed to the PBS Group (*p* = 0.0455, Cohen's *d* = −1.89). Besides, there was a somewhat higher CD31 level in the PBS Group than in the Blank Group without statistical significance (*p* = 0.6737, Cohen's *d* = −0.22) (Figure 4).

### 3.5 TWEAK stimulation regulates HG‐induced HUVECs and Fn14/EGFR signals in the wound tissue of diabetic mice

**FIGURE 5:**
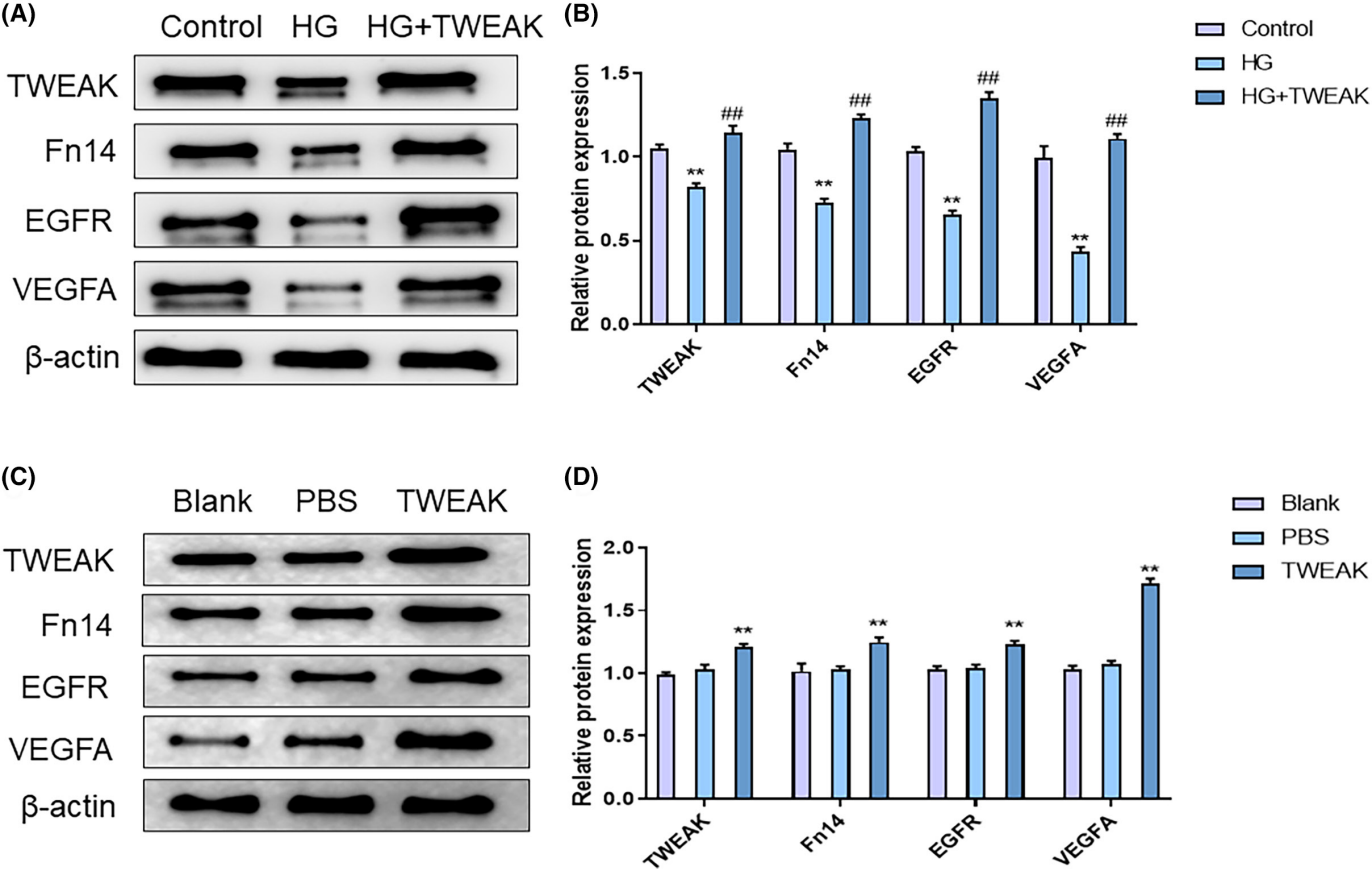
TWEAK stimulation regulates HG‐induced HUVECs and Fn14/EGFR signals in the wound tissue of diabetic mice. (A) Western blot assay was used to detect the protein levels of TWEAK, Fn14, EGFR, and VEGFA in HUVECs. (B) Relative protein expression of TWEAK, Fn14, EGFR, and VEGFA in HUVECs. **p < 0.01 versus Control Group; ^##^ *p <* 0.01 versus HG Group. (C). Western blot assay was used to detect the protein levels of TWEAK, Fn14, EGFR, and VEGFA in the wounded skin tissue of mice on day 12. (D) Relative protein expression of TWEAK, Fn14, EGFR, and VEGFA in the wounded skin tissue of mice on day 12. **p < 0.01 versus PBS Group. TWEAK, tumor necrosis factor‐like weak inducer of apoptosis; Fn14, fibroblast growth factor‐inducible 14; EGFR, epidermal growth factor receptor; HUVECs, human umbilical vein endothelial cells; H&E, hematoxylin–eosin; PBS, phosphate buffer saline.

Researches disclosed that TWEAK could stimulate the expression of EGFR and VEGFA. In vitro, Western blot results demonstrated a notable decline in the protein levels of TWEAK (*p* = 0.0002, Cohen's *d* = 10.65), Fn14 (*p* < 0.0001, Cohen's *d* = 12.78), EGFR (*p* < 0.0001, Cohen's *d* = 18.14), and VEGFA (*p* < 0.0001, Cohen's *d* = 11.12) in the HG Group in contrast to the Control Group. However, these protein levels increased notably in the HG + TWEAK Group as opposed to the HG Group (*p* < 0.0001, Cohen's *d* = −11.00; *p* < 0.0001, Cohen's *d* = −21.97; *p* < 0.0001, Cohen's *d* = −28.25; *p* < 0.0001, Cohen's *d* = −26.27) (Figure 5A,B). In vivo, these protein levels were remarkably upregulated in the TWEAK Group relative to the PBS Group on day 12 (*p =* 0.0010, Cohen's *d* = −6.71; *p =* 0.0025, Cohen's *d* = −5.71; *p* = 0.0003, Cohen's *d* = −7.61; *p* < 0.0001, Cohen's *d* = −26.34), whereas there was no significant difference (*p =* 0.2714, Cohen's *d* = −1.76; *p =* 0.9465, Cohen's *d* = −0.24; *p =* 0.9363, Cohen's *d* = −0.32; *p =* 0.2876, Cohen's *d* = −1.76) between the PBS Group and Blank Group (Figure 5C,D).

## 4 DISCUSSION

The in vitro experiments in this study revealed that TWEAK encouraged the proliferation, migration, and tubule formation in HG‐induced HUVECs. Moreover, the local application of TWEAK was shown to accelerate the skin wound healing of diabetic mice, primarily through enhanced CD31 expression which supports angiogenesis, as well as through the TWEAK/Fn14 signaling promoting epidermal tissue regeneration and neovascularization formation. Our results powerfully affirmed the role of TWEAK/Fn14 signaling in mediating the skin wound healing process in diabetic mice.

TWEAK has been proven to be essential for wound repairing because it can regulate inflammatory response, fibrosis, and tissue remodeling via its interaction with Fn14.^16^ Briefly, TWEAK and Fn14 can be bound and activated on the skin lesion surface, thereby regulating the proliferation and differentiation of fibroblasts and endothelial cells as well as promoting the granulation tissue formation, neovascularization formation, and re‐epithelialization. Moderate activation of Fn14 with TWEAK preparation is beneficial to facilitate the repair of acute skin wounds.^7^ Also, external application of TWEAK can promote the burn wound healing in wild‐type mice, but it has no significant effect on Fn14‐deficient mice. Concerning the wounds of wild‐type mice, TWEAK also promotes the infiltration of inflammatory cells and increases the generation of extracellular matrix components and growth factors.^19^ Consistent with these findings, our study observed downregulated TWEAK expression in HG‐induced HUVECs and diabetic mice. However, the decline above were all effectively recovered after TWEAK treatment, further implicating the contribution of TWEAK/Fn14 in skin repair.

Angiogenesis exerts crucial functions in promoting would repair because it allows oxygen and nutritious substances to reach the wounded sites.^20^ Impaired angiogenesis is intimately associated with dysfunctional endothelial cells, which is a key feature of diabetes and a major contributor to impaired wound healing in diabetes.^21^, ^22^ EGFR signaling pathway participates in multiple biological processes, including angiogenesis, by influencing the proliferation, apoptosis, and migration of the endothelial cells.^23^ Previous studies have pointed out that, HG level impairs the transduction of EGFR signals, consequently delaying wound healing.^24^ Consistent with the above findings, this study showed that EGFR expression was downregulated both in HG‐treated HUVECs and diabetic mouse skin wounds.

VEGFA is an important growth factor that participates in the skin wound healing. VEGFA‐mediated signaling cascades can regulate the ability of vascular endothelial cells to proliferate, migrate, survive, and angiogeneize.^25^, ^26^ Studies indicate that HG level inhibits VEGFA signal transduction, further contributing to the prolonged healing duration of wounds.^27^ Furthermore, other studies have demonstrated that TWEAK also regulates VEGFA expression.^12^ This study corroborates these findings by disclosing that VEGFA expression was downregulated in both HG‐treated HUVECs and diabetic mice skin wound. Moreover, our findings reveal a regulatory role of TWEAK in the expression of VEGFA, which can promote wound healing when activated. In all, our results indicated that the activation of TWEAK/Fn14 signaling appears to stimulate angiogenesis and plays a beneficial role in the healing of diabetic wounds.

Despite the significant findings, our study has several limitations. Firstly, the mechanistic pathways by which TWEAK enhances VEGFA expression and its precise role in angiogenesis require further elucidation. Therefore, further studies should focus on exploring the detailed molecular mechanisms underlying TWEAK‐mediated VEGFA upregulation, considering other potential signaling pathways or regulatory mechanisms involved. This comprehensive approach helps to avoid confirmation bias and provides a more holistic understanding of TWEAK's role in angiogenesis. Secondly, our study was limited to HG‐treated HUVECs and diabetic mouse models, which may not fully represent the complexity of diabetic wound healing in humans. It would be beneficial to investigate the role of TWEAK in other cell types and diabetic models to validate and extend our findings. Thirdly, the duration and dosage of TWEAK treatment need optimization to evaluate its therapeutic potential accurately. Future research should systematically investigate the effects of different dosages and treatment duration to determine the optimal therapeutic regimen.

## 5 CONCLUSION

This study underscores the importance of EGFR and VEGFA signaling in angiogenesis and wound healing in diabetic conditions and highlights the therapeutic potential of targeting the TWEAK/Fn14 pathway. Continued research in this area may lead to novel interventions that improve healing outcomes for patients with diabetes.

## AUTHOR CONTRIBUTIONS

**Ying‐jie Zhu**: Conceptualization; Investigation; Roles/Writing—original draft. **Hu‐lin Chen**: Data curation; Formal analysis. **Jing‐kai Huang**: Project administration; Resources. **Xin‐jie Cai**: Methodology; Writing—review and editing. **Bang‐le Zhan**: Software; Supervision.

## FUNDING INFORMATION

This study was supported by Southern University of Science and Technology Hospital Director's Research Fund (No. 2021‐C5).

## CONFLICT OF INTEREST STATEMENT

The authors declare no conflicts of interest.

## ETHICS STATEMENT

The animal experiments described in this study were authorized by the Committee of the Guangdong Medical Laboratory Animal Center Laboratory (Identification number: D202311‐9) and conducted in compliance with the institutional guidelines (Directive 2010/63/EU in Europe) for the care and use of animals.

## Data Availability

The data that support the findings of this study are available from the corresponding author upon reasonable request.
