# Correction to “TWEAK increases angiogenesis to promote diabetic skin wound healing by regulating Fn14/EGFR signaling”

**DOI:** 10.1111/jocd.70721

**Published:** 2026-02-05

Zhu YJ, Chen HL, Huang JK, Cai XJ, Zhan BL. TWEAK increases angiogenesis to promote diabetic skin wound healing by regulating Fn14/EGFR signaling. J Cosmet Dermatol. 2024;23(12):4230–4238.

In Figure 3C, the specific cause was determined to be an error in file naming and batch export parameter settings during data processing and image export.

The revised version of Figure 3 is shown below.

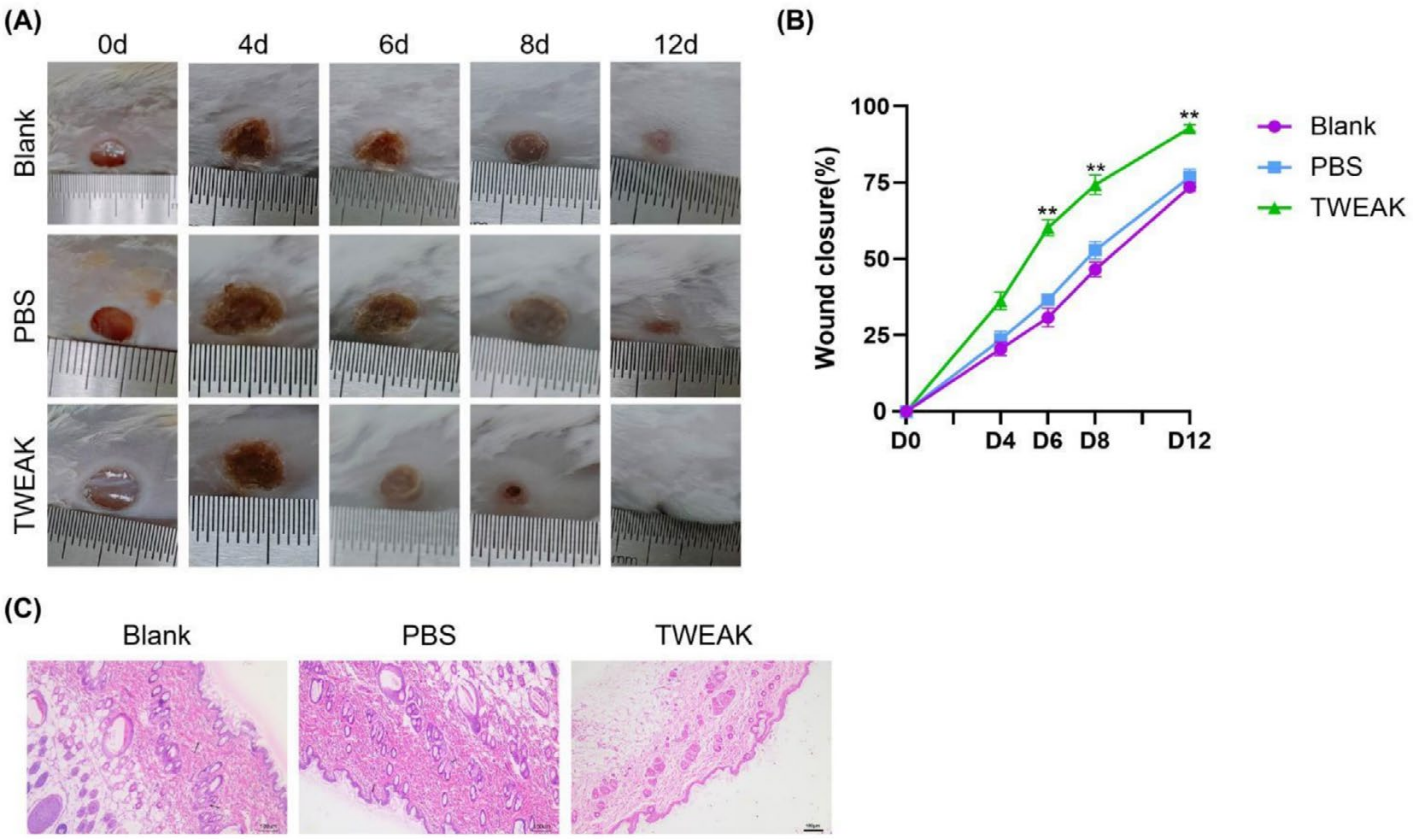

We apologize for this error.

